# Exploring the landscape of drug resistance in gastrointestinal cancer immunotherapy: A review

**DOI:** 10.1097/MD.0000000000036957

**Published:** 2024-01-12

**Authors:** Nan Yao, Wenqiang Li, Ning Duan, Guoshuai Xu, Guoyong Yu, Jun Qu

**Affiliations:** a Department of General Surgery, Aerospace Center Hospital, Beijing, China; b Department of Nephrology, Beijing University of Chinese Medicine Affiliated Dongzhimen Hospital, Beijing, China.

**Keywords:** cancer immunotherapy, drug resistance, ferroptosis, immune checkpoint blockade, tumor microenvironment

## Abstract

Gastrointestinal (GI) cancers pose a significant challenge due to high prevalence and mortality. While advancements in detection and conventional treatments have been made, prognosis often remains poor, particularly for advanced-stage cancers. Immunotherapy has emerged as a transformative approach, leveraging the body immune system against cancer, including immune checkpoint inhibitors (ICIs), cancer vaccines, and adoptive cell transfer. These modalities have shown promise, achieving sustained responses and improved survival in some patients. However, their efficacy in GI cancers is less pronounced, hindered by drug resistance mechanisms that are either intrinsic or acquired over time. This review examines the latest understanding of immunotherapy in GI cancers, focusing on ICIs, cancer vaccines, and adoptive cell transfer, along with their associated outcomes and limitations. It delves into the mechanisms behind drug resistance, including alterations in immune checkpoints, the immunosuppressive tumor microenvironment, and genetic/epigenetic changes. The role of the gut microbiome is also considered as an emerging factor in resistance. To combat drug resistance, strategies such as enhancing immune response, targeting the tumor microenvironment, and modulating resistance mechanisms are explored. The review underscores the potential of ferroptosis induction as a novel approach. Looking forward, it highlights the need for personalized immunotherapies, understanding the influence of the gut microbiome, and further exploration of ferroptosis in overcoming resistance. While challenges persist, the continuous evolution in GI cancer immunotherapy research promises innovative treatments that could significantly improve patient outcomes.

## 1. Introduction

Gastrointestinal (GI) cancers, which assail the digestive tract, have a significant presence in the global cancer landscape due to their prevalence and mortality rates.^[1]^ This group includes cancers of the esophagus, stomach, liver, pancreas, gallbladder, and colorectum. Despite advancements in early detection and treatments such as surgery, radiation, and chemotherapy, the prognosis remains poor for many, especially those diagnosed at advanced stages.^[2,3]^ In recent times, immunotherapy has risen to the forefront of cancer treatments, offering hope for numerous cancer types, including GI cancers.^[4]^ Immunotherapy harnesses the body immune system to detect and eliminate malignant cells. Its range of therapies includes immune checkpoint inhibitors (ICIs), cancer vaccines, and the innovative method of adoptive cell transfer (ACT).^[5–7]^ These treatments have yielded promising outcomes in various cancers, such as lung cancer,^[8,9]^ breast cancer,^[10]^ and melanoma,^[11,12]^ resulting in sustained responses and enhanced survival for some patients. However, the effectiveness of immunotherapy for GI cancers has not been as pronounced, especially when compared to its notable successes in treating cancers like melanoma and lung cancer.^[13]^ A significant challenge is the development of drug resistance, where cancer cells cleverly adapt, continuing to grow, and spread despite immunotherapeutic interventions.^[14,15]^ This resistance can be present from the start of treatment (intrinsic resistance) or may develop as treatment progresses (acquired resistance).^[16]^ It’s crucial to understand the mechanisms of drug resistance in GI cancer immunotherapy. This understanding helps predict patient responses to treatment and drives the development of innovative strategies to overcome resistance. This review highlights the current understanding of immunotherapy in GI cancer, drug resistance mechanisms in GI cancer immunotherapy, with a focus on the role of methods in overcoming drug resistance. The review also discusses current strategies and future directions in overcoming drug resistance in GI cancer immunotherapy.

## 2. Immunotherapy in gastrointestinal cancer

### 2.1. Immune checkpoint inhibitors and GI cancers

In the realm of GI cancers, inhibitors targeting PD-1 and PD-L1 stand out as the most prevalent immune checkpoint modulators. A study focused on the safety of anti-PD-1/PD-L1 immunotherapy highlighted its potential in patients diagnosed with gastric esophageal cancer, emphasizing the long-term survival outcomes.^[17]^ Another investigation into pembrolizumab, a PD-1 inhibitor, underscored its efficacy in gastric cancer, especially in the context of PD-L1 expression.^[18]^ Comparative analyses have shown varying effectiveness of these inhibitors in treating esophageal or gastric/gastroesophageal junction cancer.^[19]^ Furthermore, a comprehensive review indicated that PD-1/PD-L1 inhibitors could significantly prolong the overall survival (OS) of advanced gastroesophageal cancer patients.^[20]^ A pooled analysis from various clinical trials revealed that PD-1/PD-L1 inhibitors had a mean overall response rate of 19.56%, with a median time to response of 2.05 months and a median duration of response of 10.65 months.^[21]^ Nevertheless, these findings underscore the transformative potential of ICIs in the management of GI cancers, though challenges remain in optimizing patient selection and overcoming resistance (Table 1).

**Table 1:** The relevant studies linking the relationship between immunotherapy and gastrointestinal cancer.

Study	Design	Samples, n	Conclusions
Chen et al^[17]^	Review and meta-analysis	2084 participants	Anti-PD-1/PD-L1 immunotherapy has demonstrated superior therapeutic efficacy in treating advanced gastric esophageal cancer compared to chemotherapy or palliative care
Brar and Shah^[18]^	Review	NA	Pembrolizumab is a promising treatment for gastric cancer, but more research is needed to determine which patients will benefit from it by studying molecular tumor characteristics and the immune microenvironment
Oh et al^[19]^	Review and meta-analysis	4206 participants	The use of PD-1/PD-L1 inhibitors has shown improved overall survival and progression-free survival rates
Gu et al^[20]^	Meta-analysis of RCTs	9304 participants	PD-1/PD-L1 inhibitors could prolong the OS of advanced gastroesophageal cancer patients
Chen et al^[21]^	Meta-analysis	16,400 participants	High sodium intake is strongly and independently associated with an increased risk of cardiovascular disease and all-cause mortality in overweight persons
Liu et al^[22]^	Review	NA	Identifying neoantigens, developing combination therapy, and optimizing vaccine platforms are crucial in the development of cancer vaccines as a potent strategy in immunotherapy for solid tumors
Dayoub and Davis^[23]^	Review	NA	Therapeutic vaccines for cancer may have greater benefits for patients in the future if they are better integrated with other treatment options
Tay et al^[24]^	Review	NA	Combining antigen-based therapies with new drugs is a precise approach and could be the future of cancer vaccines
Chudasama et al^[25]^	Review	NA	Novel agents, including cancer vaccines, have been developed as a result of improved understanding of the interaction between the immune system and tumors
Enokida et al^[26]^	Review	NA	Preventing cancer through vaccines has shown greater progress than eliminating established cancer, especially in the case of vaccines targeting oncogenic viruses
Vasen et al^[27]^	Review	NA	The clinical management of affected families can be significantly improved with the help of hereditary cancer registries, with the Lynch syndrome registry playing a crucial role in this regard
June^[28]^	Review	NA	To be commercially viable, adoptive T cell therapy must be clinically effective, scalable, reproducible, and appropriately priced and marketed
Rosenberg et al^[29]^	Review	NA	Recent genetic engineering breakthroughs have expanded the potential of ACT immunotherapy, offering a promising new approach to treating diverse cancer types
Kirtane et al^[30]^	Review	NA	While ACT has revolutionized the treatment of blood cancers, its application in treating solid tumors is still in the early stages of development
Morotti et al^[31]^	Review	NA	The mining of exomic sequencing data has played a crucial role in the identification of mutated antigens that are recognized by tumor-reactive T cells following adoptive transfer
Parkhurst et al^[32]^	Clinical trail	8 participants	Combining adoptive transfer of NK cells with monoclonal antibody administration deserves further evaluation as a potential therapy, as persistent NK cells can induce antibody-dependent cell-mediated cytotoxicity without cytokine reactivation in vitro

ACT = adoptive cell therapy, NK = natural killer, OS = overall survival.

### 2.2. Cancer vaccines and GI cancers

Cancer vaccines, designed to stimulate the body immune system to target and destroy cancer cells, represent a promising avenue in the realm of solid tumor immunotherapy.^[22]^ Specifically, within the domain of GI cancers, there’s a discernible correlation between the annual incidence of cancer and the ongoing activities in therapeutic cancer vaccine trials.^[23]^ The journey of cancer vaccine development has spanned over 5 decades, and while there have been significant strides, the overall success has been moderate.^[24]^ Epidemiological studies have provided insights into the challenges and potential of cancer vaccines in GI cancers. For instance, earlier studies from the 1970s investigated the possibility of targeting specific cancer antigens as a therapeutic approach for GI cancers.^[25]^ Furthermore, research has indicated that therapeutic cancer vaccine clinical trials correspond with disease incidence in the US, but not necessarily with measures of mortality.^[23]^ Another intriguing aspect is the exploration of tumor antigen-based vaccines, which have demonstrated the ability to prevent cancer in mouse models.^[26]^ Additionally, ongoing studies are focusing on preventing or delaying cancer in individuals with genetic predispositions, such as Lynch syndrome^[27]^ (Table 1).

### 2.3. Adoptive cell transfer and GI cancers

ACT is a therapeutic approach where immune cells are isolated, expanded, and then reintroduced into a patient to target cancer cells.^[28]^ In the realm of GI cancers, ACT has shown potential in clinical trials. One landmark study demonstrated a 55% objective response rate using the adoptive cellular transfer of tumor-infiltrating lymphocytes.^[29]^ Additionally, advancements in genetic engineering have enabled the modification of normal human lymphocytes to recognize specific cancer antigens, leading to observed cancer regression in vivo.^[30]^ Mining exomic sequencing data has also been instrumental in identifying mutated antigens recognized by adoptively transferred tumor-reactive T cells.^[31]^ Experimental designs have explored the potential of ACT using in vitro activated autologous natural killer (NK) cells in patients with metastatic melanoma or renal cell carcinoma.^[32]^ As the field of ACT continues to evolve, its potential in addressing GI malignancies remains a significant area of research (Table 1).

## 3. Mechanisms of drug resistance in immunotherapy

**Figure 1.**
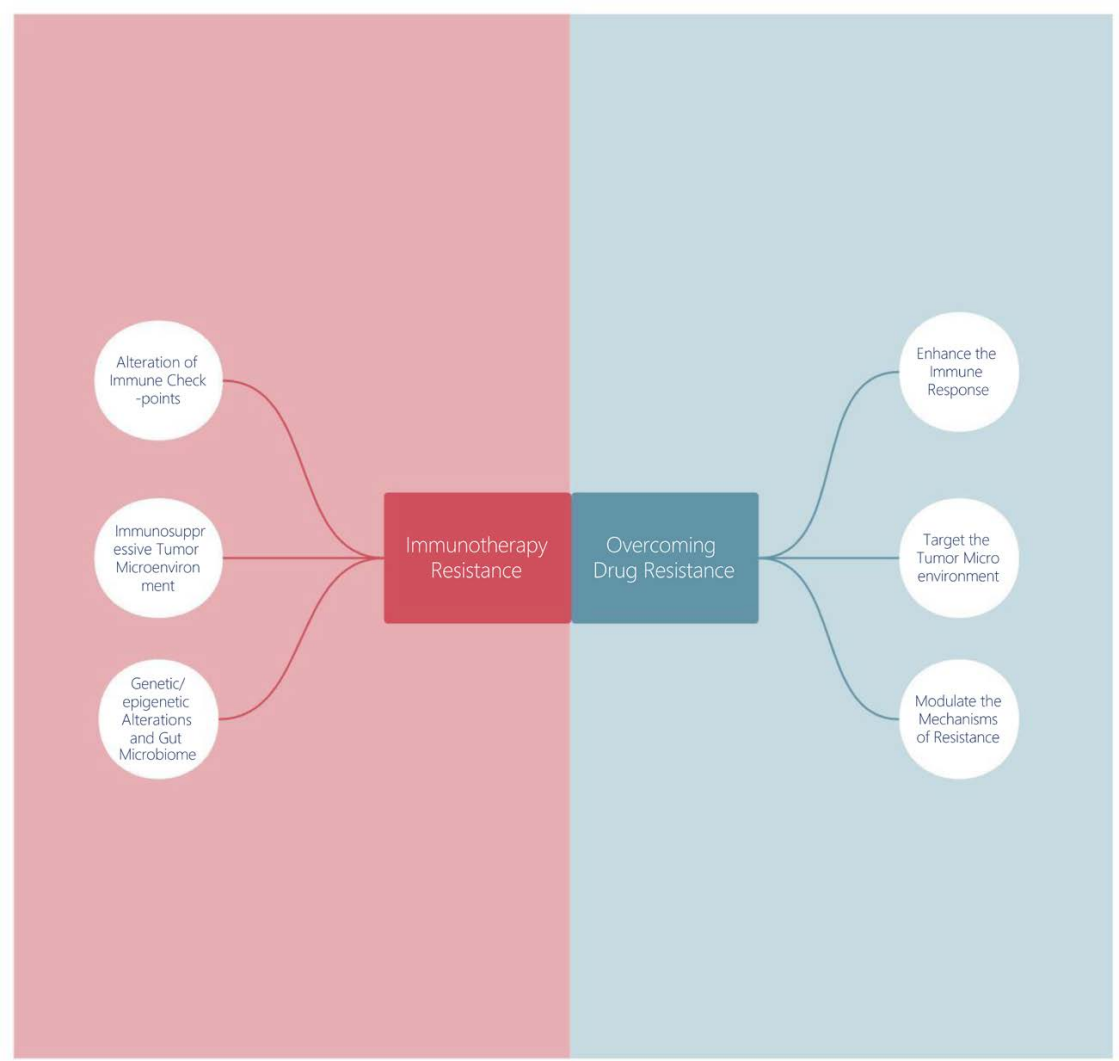
The mechanisms of drug resistance in gastrointestinal cancer immunotherapy and related strategies.

Immunotherapy, a groundbreaking strategy that mobilizes the body immune defenses to counteract cancer, has demonstrated substantial potential in the management of diverse malignancies. However, its application in GI cancers has been met with substantial challenges, one of the most formidable being drug resistance. This resistance can manifest in 2 forms: intrinsic and acquired. Intrinsic resistance refers to the initial unresponsiveness of the tumor to the therapy, while acquired resistance develops over time after an initial response.^[33]^ Both forms of resistance pose significant hurdles to the successful treatment of GI cancers with immunotherapy. An in-depth comprehension of the mechanisms underpinning these forms of resistance is crucial for enhancing immunotherapy efficacy and developing strategies to overcome resistance (Fig. 1).

### 3.1. Alteration of immune checkpoints

One of the primary mechanisms of resistance is the alteration of immune checkpoints.^[34]^ Immune checkpoints are regulatory pathways integral to maintaining self-tolerance and modulating immune responses to prevent tissue damage. Tumors can manipulate these checkpoints, upregulating the expression of immune checkpoint proteins such as PD-L1, effectively stifling the immune response.^[35]^ This manipulation allows tumor cells to evade immune surveillance and continue to proliferate, despite the presence of immunotherapeutic agents. In some instances, tumors can also develop mutations that render them resistant to the effects of ICIs.^[36]^

### 3.2. Immunosuppressive tumor microenvironment

Another significant mechanism of resistance is the creation of an immunosuppressive tumor microenvironment. Tumors can create an environment hostile to immune cells, making it difficult for these cells to survive and function effectively.^[37]^ This adverse milieu can be engineered through the enlistment of immunosuppressive cells, including regulatory T cells and myeloid-derived suppressor cells, coupled with the secretion of immunosuppressive cytokines like transforming growth factor beta and interleukin-10.^[38]^ These factors collectively contribute to a microenvironment that hinders the immune response and promotes tumor growth.

### 3.3. Genetic/epigenetic alterations and gut microbiome

Genetic and epigenetic alterations in tumor cells also play a significant role in resistance. These alterations can affect the expression of tumor antigens, making it difficult for immune cells to recognize and target the tumor cells. They can also affect the signaling pathways involved in the immune response, leading to an impaired immune response.^[39,40]^ Recently, the gut microbiome has emerged as a potential player in immunotherapy resistance. Certain gut bacteria have been associated with resistance to immunotherapy, possibly by modulating the immune response.^[41,42]^ This represents a relatively nascent field of investigation, necessitating further studies to comprehensively elucidate the role of the gut microbiome in resistance to immunotherapy.

## 4. Overcoming drug resistance in GI cancer immunotherapy

The challenge of drug resistance in GI cancer immunotherapy is a significant hurdle that necessitates the development of innovative strategies to enhance treatment efficacy. The strategies to combat this resistance can be broadly categorized into 3 main areas: those that aim to enhance the immune response, those that target the tumor microenvironment, and those that modulate the mechanisms of resistance (Fig. 1).

### 4.1. Enhance the immune response

Amplifying the immune response is a cornerstone strategy in addressing drug resistance in oncology, with particular relevance to GI cancers.^[43]^ Effective activation of the immune system enables the identification and targeting of malignant cells, offering a potential avenue to overcome the challenges of drug resistance, a significant hurdle in the treatment of GI malignancies.^[36]^ To this end, a plethora of innovative methodologies have been devised, with combination therapies emerging as a standout approach. These therapies amalgamate multiple therapeutic agents to concurrently target diverse pathways, thereby diminishing the propensity of malignancies, especially GI tumors, to develop resistance.^[44]^ ICIs are a testament to modern advancements in this domain and have shown promising results in preclinical models and clinical settings for GI cancers. These agents obstruct proteins that deter immune cells from assailing cancerous cells. Nevertheless, resistance to ICIs can manifest, prompting the investigation of their confluence with other therapeutic modalities, such as chemotherapy, radiation, or targeted therapies, to mitigate this challenge.^[45]^ A prime example of such investigative efforts is the ongoing single-arm phase II trial (JapicCTI-205400),^[46]^ which is evaluating the efficacy and safety of nivolumab combined with low-dose ipilimumab in patients with microsatellite instability-high metastatic Gastric/Gastroesophageal Junction Cancers. This trial is a testament to the potential of combination regimens in GI cancers, aiming to bolster the immune response and assail the oncogenic growth and survival mechanisms from varied dimensions. These combined regimens not only bolster the immune response but also assail the oncogenic growth and survival mechanisms from varied dimensions. Moreover, meticulous crafting of treatment schedules is paramount. An optimized regimen ensures judicious drug administration, balancing therapeutic advantages with side effect mitigation.^[45]^

### 4.2. Target the tumor microenvironment

Targeting the tumor microenvironment is emerging as a pivotal strategy, especially in the context of GI cancers where drug resistance poses a significant challenge.^[47]^ The tumor microenvironment, characterized by its complex cellular and molecular composition, plays a crucial role in mediating resistance to immunotherapy.^[48]^ Within this environment, various factors can create a hostile milieu for immune cells, hindering their survival and effective functioning. This hostile environment often results from the presence of immunosuppressive cells and factors that can dampen the immune response.^[49]^ Strategies that specifically target and modulate the tumor microenvironment are gaining traction in the field of GI cancer immunotherapy. For instance, the use of anti-angiogenic agents can disrupt the blood supply to the tumor, thereby weakening its growth and survival.^[50]^ Additionally, inhibitors that target immunosuppressive cells, such as regulatory T cells and myeloid-derived suppressor cells, can potentially rejuvenate the immune response within the tumor microenvironment.^[51]^ By employing these strategies, the aim is to reshape the tumor milieu, making it more conducive for immune cells to operate effectively.

### 4.3. Modulate the mechanisms of resistance

Modulating the mechanisms of resistance, such as the induction of ferroptosis, is emerging as a cutting-edge strategy in the realm of cancer immunotherapy, particularly in GI cancers.^[52]^ Ferroptosis is a form of regulated cell death characterized by iron-dependent lipid peroxidation, and its role in the progression of GI cancers is becoming increasingly recognized. This process influences not only the proliferation of cancer cells but also their invasive and metastatic potential. Studies have identified genes like “GABPB1-AS1”^[53]^ and “SLC7A11”^[54]^ in hepatocellular carcinoma as pivotal regulators of ferroptosis, indicating their significant impact on the disease progression and response to therapy. The intricate relationship between ferroptosis and immunotherapy resistance in GI cancers is complex and not yet fully understood. Preliminary studies have shown that cancer cells may evade immunotherapeutic interventions by modulating ferroptotic pathways. For instance, “APOC1” has been demonstrated to enhance the efficacy of anti-PD1 immunotherapy in patients with hepatocellular carcinoma by influencing the ferroptosis pathway, which leads to the conversion of immunosuppressive M2 macrophages to pro-inflammatory M1 macrophages, thereby augmenting the immune response.^[55]^ Thus, harnessing the potential of ferroptosis might offer a novel approach to enhance the responsiveness of GI tumors to immunotherapy. Yet, it’s imperative to tread with caution. While inducing ferroptosis in cancer cells holds therapeutic promise, the safety and efficacy of such interventions need rigorous validation. As the field of ferroptosis continues to evolve, it’s paramount that researchers delve deeper into its mechanistic underpinnings, especially in the context of GI cancer immunotherapy, to ensure the development of safe and effective therapeutic strategies.^[56]^

## 5. Future perspectives

The field of GI cancer immunotherapy is rapidly evolving, with continuous investigations focusing on deciphering the intricacies of drug resistance and devising innovative approaches to overcome this hurdle.^[57]^ The future of this field holds promise, but also presents challenges that need to be addressed. One of the potential future directions in this field is the development of personalized immunotherapies. Given the heterogeneity of GI cancers and the variability in patient responses to immunotherapy, personalized approaches that consider the unique characteristics of each patient tumor could potentially enhance treatment efficacy and overcome resistance.^[58]^ This could encompass the utilization of predictive biomarkers to pinpoint patients who are likely to respond favorably to immunotherapy, or the development of personalized cancer vaccines based on the unique mutational landscape of each patient tumor.

Another burgeoning area of research is the potential influence of the gut microbiome in shaping the resistance to immunotherapy.^[59]^ Recent research has illuminated the possibility that specific microbiota residing in the gut may have the capacity to shape immune responses, thereby influencing the effectiveness of immunotherapy. Understanding the complex interactions between the gut microbiome and the immune system could potentially unlock novel strategies for microbiome modulation, thereby enhancing the efficacy of immunotherapy.

The prospective role of ferroptosis in overcoming drug resistance is another exciting area of research.^[60]^ As previously expounded, ferroptosis represents a regulated cell death modality, driven by iron-catalyzed lipid peroxidation. Although the function of ferroptosis in immunotherapy resistance remains partially obscured, current research endeavors in this field hold the potential to engender the development of novel therapeutic strategies that induce ferroptosis and surmount resistance. However, these potential future directions also present challenges. For example, the development of personalized immunotherapies requires a deep understanding of the complex biology of GI cancers and the immune system, as well as the development of robust methods for the identification and validation of predictive biomarkers. Similarly, the modulation of the gut microbiome or the induction of ferroptosis could have potential side effects that need to be carefully considered.

## 6. Conclusions

GI cancers remain a formidable challenge in the oncological arena, necessitating innovative therapeutic strategies to improve patient outcomes. Immunotherapy, with its potential to harness the body immune defenses, offers a promising avenue for the management of these malignancies. However, the specter of drug resistance looms large, underscoring the need for a comprehensive understanding of its underlying mechanisms. The interplay between the tumor microenvironment, genetic and epigenetic alterations, and external factors like the gut microbiome presents a complex landscape that influences therapeutic outcomes. The emergence of strategies targeting these facets, from personalized immunotherapies to the modulation of ferroptosis, signifies the evolving nature of this field. As we forge ahead, it is imperative to continue refining these approaches, ensuring their safety and efficacy, while also remaining vigilant to the challenges that lie ahead. The future of GI cancer immunotherapy, though replete with challenges, holds the promise of transformative treatments that could significantly alter the trajectory of this disease.

## Author Contributions

All authors have read and approved the manuscript. Nan Yao: Writing-Original draft preparation; Wenqiang Li: Writing-Reviewing and Editing; Ning Duan: Writing-Reviewing and Editing; Guoshuai Xu: Visualization; Guoyong Yu: Methodology; Jun Qu: Conceptualization, Supervision.
